# *Trichinella* Outbreaks on Pig Farms in Poland in 2012–2020

**DOI:** 10.3390/pathogens10111504

**Published:** 2021-11-18

**Authors:** Ewa Bilska-Zając, Mirosław Różycki, Weronika Korpysa-Dzirba, Aneta Bełcik, Anna Ziętek-Barszcz, Magdalena Włodarczyk-Ramus, Aneta Gontarczyk, Tomasz Cencek

**Affiliations:** 1 Department of Parasitology and Invasive Diseases, National Veterinary Research Institute, Partyzantów Avenue, 57, 24-100 Pulawy, Poland; ewa.bilska@piwet.pulawy.pl (E.B.-Z.); mrozycki@piwet.pulawy.pl (M.R.); aneta.belcik@piwet.pulawy.pl (A.B.); magdalena.wlodarczyk@piwet.pulawy.pl (M.W.-R.); aneta.gontarczyk@piwet.pulawy.pl (A.G.); tcencek@piwet.pulawy.pl (T.C.); 2 Department of Epidemiology and Risk Assessment, National Veterinary Research Institute, Partyzantów Avenue, 57, 24-100 Pulawy, Poland; anna.barszcz@piwet.pulawy.pl

**Keywords:** *Trichinella* spp., pig, Poland, outbreak, farm

## Abstract

*Trichinella* nematodes continue to circulate in various hosts both in the domestic and sylvatic cycles. In the majority of countries in Europe, wild boars have been noticed as a primary source of *Trichinella* spp. infections in humans. However, in some regions, the meat of pigs containing *Trichinella* spp. larvae can still be a cause of trichinellosis. Therefore, in the present study, we aimed to determine and present actual data on the occurrence of *Trichinella* spp. on pig farms (*Sus scrofa f. domestica*) in Poland. In this study, over 194 million pigs, slaughtered for commercial and personal purposes between 2012 and 2020, were tested with a digestion method according to the official rules for *Trichinella* control. Positive results were noticed in 172 pigs which gives an overall prevalence of 0.000088%. On seven farms, rats (*Rattus norvegicus*) infected with *Trichinella* spp. were also discovered. The species identification showed pigs were infected with *Trichinella spiralis* on 26 farms, and on four farms pigs with *Trichinella britovi* infections were found. Therefore, it is important to constantly monitor pigs for the presence of these parasites, especially in view of the growing interest in organic meat originated from ecological farms.

## 1. Introduction

Food safety is a key issue for the global food chain and a daily concern for consumers. Foods containing pathogenic bacteria, viruses, parasites or fungi pose a risk to the health and life of consumers. This increases the demand for health services, government spending on public health and other social costs. In order to prevent the above risks, the European Union has introduced quality assurance programs based on continuous monitoring of the raw material, production process, storage and distribution of final products, for the purpose for which they are intended [1]. Pig meat in Poland is one of the most consumed meats, with more than 40 kg of pork consumed per capita [2]. As a final product, it can be a source of many zoonoses, including trichinellosis [3]. Trichinellosis is caused by nematodes of the genus *Trichinella*. The most pathogenic species and the most common cause of this disease in humans is *Trichinella spiralis* [4]. In Poland, this species is responsible for the majority of infections [5]. This parasite spreads in the natural environment by consuming meat containing live larvae; therefore, carnivores and omnivoresare the most vulnerable to infection. However, herbivores may also be infected occasionally (e.g., due to their protein supplementation or accidentally) [6]. Due to their circulation between different host species, the synanthropic (domestic) and sylvatic (forest) cycles occur. In the synanthropic cycle, the hosts of the parasite are farm animals, primarily pigs, as well as various species of domestic and free-living animals, including rats, mice, cats, etc. living on or near farms. In the sylvatic cycle, there are many species of animals that can be infected with *Trichinella* spp., mainly predatory, carnivorous animals such as wolves, foxes, jackals, bears, and they are the largest reservoir of this parasite. Omnivores, especially wild boars, are lately more and more an important reservoir of *Trichinella* spp. The domestic cycle and the sylvatic cycle often overlap by common reservoirs such as rodents, living in the fields in summer, and gathering on farms in winter, where they have easy access to food. Rodents can transfer the nematodes both from the sylvatic cycle to the synanthropic cycle and vice versa. Sometimes, man’s behaviour is the cause of the parasite transmission between both cycles. This is the case when farm owners feed farmed animals with the remains of hunted animals (wild boar, foxes) or slaughter waste, and also when fallen livestock is illegally transported to the forest. In terms of safety for consumers, infections in the animal population intended for consumption, and therefore mainly pigs and wild boar, are of greatest importance. Historical data indicate that in Europe, pig meat was the most common source of human infection [7,8]. This information led to changes in the legislation stating that each pig carcass intended for the market or own purposes must be tested for the presence of *Trichinella* spp. larvae, and removed from the food chain in case of positive [9]. In 2005 the European Commission has implemented a new regulation no. 2075/2005, laying down specific rules for the official controls of *Trichinella* in meat in order to improve food safety for European consumers. Apart from pigs, this rule covered wild boars, horses, bears and coypu (EU Regulation 2017/2005) [10]. Subsequent, amendments to this regulation allowed for the possibility of not performing tests on all pigs, as long as they are reared on farms that meet controlled housing conditions EU Regulation 2015/1375 [11]. However, if these conditions are not met, then there is a need to continue to perform tests according to EU Regulation 2020/1478 of 14 October 2020 [12] amending Commission Implementing Regulation (EU) 2015/1375 as regards sampling, the reference method for detection and import conditions related to *Trichinella* spp. control.

According to EU One Health Zoonoses Report (2019), the main risk factor for *Trichinella* spp. infections in domestic pigs are non-controlled housing conditions. In recent years, most human cases of trichinellosis were reported from a few countries in the eastern part of Europe and were linked to free-ranging and backyard pigs and farmed wild boar. However, taking into account the 5-year period (2015–2019), there is a decreasing trend in trichinellosis cases in Europe. The reason for this is the increasing number of pigs raised under controlled housing conditions together with the reduction of pigs not kept in these conditions, the farmer’s education as well as increased control of slaughtering of free-ranging and backyard pigs. Nevertheless, the reported number of *Trichinella*-positive domestic pigs may be underestimated as most pigs slaughtered at home are still without veterinary control [13].

Researches and statistics from recent years indicate that wild boar meat is currently the main threat of trichinellosis to Polish consumers [14], and pigs are found to be infected less often [15]. As a result, food producers more and more often are asking about the legitimacy of applying this routine control, which is extremely time-consuming and costly. However, in order for any discussions on this topic to be undertaken, one should get acquainted with the current epidemiological situation regarding this parasite.

Here, we collect and present the latest data on the occurrence of trichinellosis in pigs in Poland in 2012–2020, taking into account the geographical location of farms in which this pathogen was found. We also discuss the need of conducting proper epidemiological investigations on pig farms where *Trichinella* infected pigs were found in order to stop the spreading of this parasite and find the source of infection.

## 2. Results

Routine diagnostics of trichinellosis conducted by Veterinary Inspection Services (VIS) included 194,449,146 pigs slaughtered in 2012–2020, with average 21,605,460 pigs slaughtered yearly. The positive results come from 30 pig farms in which altogether 172 pigs were infected with *Trichinella* spp. (Table 1).

**Figure 1:**
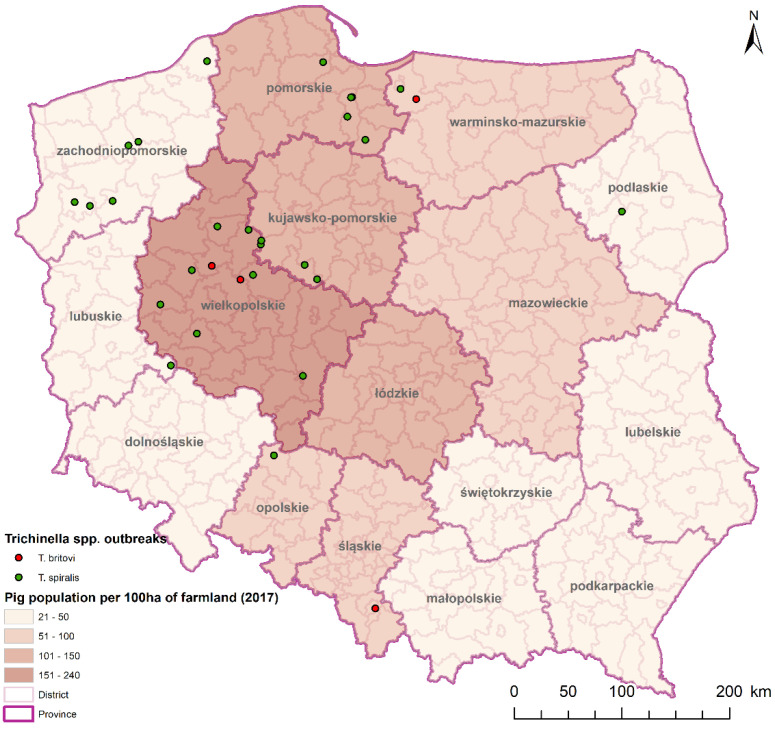
Geographical location of pig’ farms on which *Trichinella* spp. infected pigs were discovered (range of data 2012–2020). On the map pig population per 100 ha of farmland was signed by intensity of red colour.

In 18 farms, only one of the tested pigs was infected with this parasite, while in 12 farms more than two pigs were infected. Epidemiological investigations were carried out on these 12 farms. Conducted serological tests (ELISA) and confirmative digestion indicated the various amount of pigs infected with *Trichinella* spp in those 12 farms. The highest prevalence (100%) was found in 2019 on a farm (#27) where only two pigs were bred and both of them were infected. The detailed information about the number of pigs in a herd, the number of infected pigs, and the prevalence on each pig farm is shown in Table 1. Additionally, in 7 of 12 farms in which epidemiological investigation was conducted, the presence of rodents, especially rats, was noticed. On these farms, the owners collected between 3 and 56 rat carcasses. *Trichinella* spp. infected rats occurred in four farms with various prevalence. Rats from three farms were not infected with *Trichinella* spp. (Table 1). Based on geographical data, we created a map with the locations of each farm (Figure 1). The farms were located in nine provinces. The highest number of farms with infected pigs were located in Wielkopolskie (11 farms), Zachodnio-Pomorskie (six farms) and Pomorskie (five farms) voivodeship.

Species identification (using multiplex PCR) of collected isolates of larvae shows that in the majority of pigs infected with *T. spiralis* (26 farms), four pigs from four different farms were infected with *T. britovi*. Furthermore, we found:

− Moderate positive correlation between the number of tested pigs and the number of farms in which *Trichinella* spp. were detected (r = 0.45, *p* < 0.005);
− Strong positive correlation between the number of pigs in general in each province and the number of farms with infected pigs (r = 0.78, *p* < 0.005);
− Strong positive correlation between the number of pigs per 100 ha of agricultural land in each province and the number of farms with infected pigs (r = 0.86, *p* < 0.005) [16].

## 3. Discussion

In this study, we aimed to present the actual data of the occurrence of *Trichinella* spp. in pigs in Poland. The range of our study included a routine investigation for trichinellosis in pigs in the years 2012–2020. The discovered prevalence of infection in the tested pigs’ population was 0.000088%. The resulting prevalence is much lower compared to historical data, when in the years 1947–1956, 0.55% of pigs infected with *Trichinella* spp. were found, and in the following years, 1957–1963, a prevalence of 0.146% was recorded [17,18]. The other study, conducted between 1996 and 2004, showed an infection rate of 0.0054% [19]. Thus, when analyzing the obtained results over the decades, one can observe a downward trend of *Trichinella* spp. prevalence in pigs in Poland. The reason for the decrease in the percentage of pigs infected with *Trichinella* spp. is mainly the changes that have occurred in pig breeding, among others: intensification of breeding and introduction of biosecurity rules, improvement of welfare on farms, as well as introduction of industrial feed and prohibition of feeding pigs with meat and slaughter waste [20]. Despite this, Poland is one of the few European Union countries where *Trichinella* spp. in pigs is still regularly detected. In many European countries, including Austria, Belgium, Cyprus, Denmark, Ireland, Luxembourg, Malta, the Netherlands, Portugal, Slovenia, Sweden and England, no cases of pigs infected with this nematode have been reported for several years [21]. This is mainly the result of almost exclusively large-scale production and keeping animals in herds under controlled conditions. In Germany, detected cases of pig infections have been reported in recent years [22]. In the Czech Republic and Slovakia and other European Union countries, where pig production is low, pig trichinosis infections are also detected sporadically. Only in south-eastern Europe, where small farms dominate, e.g., in Bulgaria, Romania or Serbia, there are still relatively numerous infections of pigs with *Trichinella* spp. every year [23,24,25].

In the present study, almost all farms where an epidemiological investigation was conducted were small with a number of pigs less than 100. Polish pig production characterizes huge defragmentation and spread of small individual farms. The share of farms with herds of up to 200 heads in 2013 amounted to 97%, and those with herds of 200 heads or more only constituted 3% [26]. Changes in market conditions in recent decades, as well as growing requirements such as sanitary and veterinary standards, have resulted in intensive structural and modernization changes in slaughtering and pork processing. Between 2000 and 2015, the number of large companies in the meat sector increased by 25% [27]. Currently, there are about 70% of individual, non-commercial and backyard farms, while the rest are operated by companies breeding >200 pigs in a herd [26]. The structure of pig production continues to change since the African Swine Fever epidemic occurred in recent years. This epidemic caused large losses in pig production, which resulted in the liquidation of several hundred thousand small pig farms [28]. Therefore, a continuous reduction in the number of small farms for these large-scale items is expected. This trend, however, does not exclude the possibility of *Trichinella* spp. infection in such farms. There are several conditions that must be fulfilled to completely protect pigs on a farm from parasite infections [12,29]. The very important characteristics on a farm that may have an impact on the occurrence of *Trichinella* spp. infection in pigs are: presence/absence of outdoor access, type of feed and presence of rodents which may be a vector for *Trichinella* spp. In our study, the majority of farms investigated presented indoor breeding systems, but some of them have also outdoor access, at least seasonally. The epidemiological investigation showed that the welfare found on these farms leaves much to be desired. Rats were found in seven farms, and in four farms, rats were also infected with *Trichinella* spp. This result indicates possible source of infection for pigs (ingestion of rats by pigs) on the investigated farms. The presence of rats in pig farms is not one-sided, however, because rats can both transfer the parasites to the farm and then spread them to the natural environment [30]. The single incidence of pig infection on a farm (e.g., farm #4–#11) can be explained by the first scenario where a rat can be caught and eaten by pigs and then introducing parasites into the farm.

In our study, also the second scenario is possible; we suspect it especially in farms #2 and #12, where multiple infected rats were found. The epidemiological inquiry indicates that rats on these two farms were probably infected at the same time as pigs with feed contaminated by a bigger amount of fragmented, minced meat containing a relatively small number of *Trichinella* spp. larvae [31]. This conclusion we made based on epidemiological inquiry when evidence of illegal action taken by the owners, who probably used meat from hunted wild boars for pig feeding, was observed. In such a scenario, it is extremely important to conduct a thorough epidemiological investigation to stop the spreading of the parasite to the environment. Such a source of *Trichinella* spp. infection was traced during an epidemiological investigation of an outbreak in the Slovak Republic, which occurred after consumption of pork and/or smoked pork products. In this case, it was ascertained that the pig’s owner was also a hunter and occasionally fed pigs with wild boar scraps [32].

An interesting case was farm #27, in which only two pigs were bred. Animals were kept indoor in the majority, but seasonally they had access to the outdoors. On this farm, both pigs were infected with *Trichinella* spp. and each of the three trapped rats was infected too. It is extremely difficult to determine the source of infection on this farm; however, it seems probable that pigs acquired the infection outdoors. Similar findings were obtained during the epidemiological study of the outbreak which occurred in Bulgaria where *T. spiralis* larvae were detected in a brown rat (*Rattus norvegicus*) trapped near the small farm where the pigs were raised in the backyard. Consumption of those pigs’ meat resulted in trichinellosis in eight people inhabiting the farm. Therefore, also in this outbreak, it is highly possible that the rats could be the source of *Trichinella* spp. infection in pigs [33]. A recent study of pigs reared under controlled conditions in Italy, with sows having outdoor access, indicates that animals being outside has a high probability of exposure to *Trichinella* spp. [20]. This observation is not new, but it does show how important it is to monitor pigs for *Trichinella* spp. presence, even in indoor farming conditions. This fact is even more important in case of the increasing number of organic farms, where pigs have access to the external environment, especially knowing that the undercooked pork meat from free-range pig farms has already been a source of *Trichinella* spp. infection. In such a situation, the importance of the presence of *Trichinella* spp. in wild animals increases [34]. The provinces (Wielkopolskie, Zachodniopomorskie and Pomorskie) where we found the highest number of pig farms with infected animals are characterized by a high number of wild boars infected with *Trichinella* spp. [35]. These regions are also characterized by the high number of pig farms, the high density of pigs on 100 h of agricultural lands, and the high number of pigs in general [16,36]. These variables indicate a statistically significant positive correlation with the number of farms in which infected pigs were discovered. It indicates the higher risk of *Trichinella* spp. infection in areas with a higher density of pig production. This, together with the high number of infected animals in the sylvatic cycle, increases that risk. Moreover, in our study, we discovered *T. britovi* in pigs on four farms. This highlights the possibility of getting the infection from the natural environment, as this species of *Trichinella* occurs in Poland mostly in wild carnivores [15].

During epidemiological investigations on each farm, we concluded that finding the source of infection in pig outbreaks is much more complicated, and is based on suspicions more than on true evidence. The presence of rats infected or illegal feeding of pigs with leftovers from hunting is suspected to be the cause of these infections. However, there is a huge difficulty in indicating when a proper source occurs, especially when the suspected source and pigs are infected with the same *Trichinella* species, which is what occurred in most of the present study. Thus, the species identification method alone is not enough. Better tools would help control the spread on farms [35,37], as would other types of parasites [38]. Such methods will be a chance to find a source and stop further spreading of the parasite. Accurate and timely tracing of sources would aid efforts to understand and control the sources of human exposure.

## 4. Materials and Methods

The prevalence of *Trichinella* spp. in pigs was assessed based on the results of official post-mortem examinations of carcasses according to EU Regulation 2015/1375, Annex I, Chapter III. Routine diagnosis was provided by accredited field laboratories of the VIS, which used the validated reference magnetic stirrer method for pooled sample digestion to detect *Trichinella* spp. (1375/2015). Muscle samples (diaphragm and/or intercostal muscles) from pigs that tested positive for *Trichinella* spp. were provided to the National Veterinary Research Institute, which is a National Reference Laboratory for trichinellosis in Poland (NVRI) to confirm the presence of larvae and to identify their species.

The origin of each infected pig was acquired by VIS. In the case where on a farm more than one pig was infected with *Trichinella* spp., epidemiological investigation on the given farm was conducted. The epidemiological investigation consisted of several actions taken together by the NVRI and regional VIS. First, we acquired information about the herd: size, feeding system, access to the natural environment, presence of rodents. Then, we collected rat carcasses obtained by owners from farms on which rodents were present. Muscles of rats were tested in NVRI for *Trichinella* spp. presence using the same digestion method according to EU Reg. 2015/1375. Then, we collected sera from each pig on a farm and we tested them using commercial ELISA tests [39]. In case of a positive serological result, the pigs from which the blood samples were obtained were slaughtered. Then, the diaphragm muscles from their carcasses were collected and tested with the digestion method to confirm *Trichinella* spp. infection and collect larvae for species identification. Species identification was provided in NVRI using multiplex PCR according to Zarlenga et al. [40] for five randomly collected individual larvae per isolate.

### Statistical Analysis

The correlation between the number of tested pigs in each province and number of farms with infected pigs, number of pigs in general in each province and the number of farms with infected pigs, number of pigs per 100 ha of agricultural land in each province and number of farms with infected pigs were assessed by calculation of Pearson coefficient.

## 5. Conclusions

The increasingly rare finding of *Trichinella* spp. in pigs indicates the good effects of the changes that have occurred in the structure of pig production and the introduction of official regulations regarding the obligation to test the meat of pigs for *Trichinella* spp. The measurable effects of these activities include reports of the absence of cases of trichinellosis in humans caused by the consumption of infected pig meat. However, the data presented in this paper on the occurrence of pig infections in recent years indicate the need to continue the monitoring and routine tests for the presence of *Trichinella* spp. in this species of animals. Observations made during epidemiological investigations indicate the need to improve breeding conditions and broadly understand animal welfare in small individual farms, which are still the majority in Poland. This fact will become more important in the case of the increasing number of organic farms with the access of animals to the natural environment.

Epidemiological investigations have provided important information concerning possible transmission routes of *Trichinella* spp. However, the available tools were not always sufficient to properly determine the source of the parasites in a given outbreak. Therefore, there is a need to develop methods that can become a useful tool in the practical tracking of *Trichinella* spp. transmission.

## Figures and Tables

**Table 1:** List of farms in which *Trichinella* spp. infected pigs were discovered between 2012 and 2020. The table includes information about geolocation, prevalence, number of rats collected on the farm, number of infected rats and species identification of larvae.

No.	Year	Farm Location	Province	No of Pig in a Herd	No of Infected Pig	Prevalence	No of Collected Rats	No of Infected Rats	Access to Natural Environment	Species of *Trichinella*
1.	2012	Oleszno	wielkopolska	40	2	5	None	None	Yes	*T. spiralis*
2.	2013	Jeziora Wielkie	kujawsko- pomorskie	36	18	50	56	17	Yes	*T. spiralis*
3.	2013	Sliwowo- Łopienite	podlaskie	83	24	28.9	14	1	Yes	*T. spiralis*
4.	2013	Elbląg	warmińsko- mazurskie	Nd	1	Nd	Nd	Nd	Nd	*T. britovi*
5.	2013	Gniezno	wielkopolska	Nd	1	Nd	Nd	Nd	Nd	*T. britovi*
6.	2013	Damasławek	wielkopolska	Nd	1	Nd	Nd	Nd	Nd	*T. spiralis*
7.	2013	Kościan	wielkopolska	Nd	1	Nd	Nd	Nd	Nd	*T. spiralis*
8.	2013	Pyrzyce	zachodnio- pomorskie	Nd	1	Nd	Nd	Nd	Nd	*T. spiralis*
9.	2014	Rzadkwin	kujawsko- pomorskie	Nd	1	Nd	Nd	Nd	Nd	*T. spiralis*
10.	2014	Kwidzyń	pomorskie	Nd	1	Nd	Nd	Nd	Nd	*T. spiralis*
11.	2014	Kalisz	wielkopolska	Nd	1	Nd	Nd	Nd	Nd	*T. spiralis*
12.	2014	Rynowo	zachodnio- pomorskie	20	11	55	21	3	Yes	*T. spiralis*
13.	2015	Tczew	pomorskie	Nd	1	Nd	Nd	Nd	Nd	*T. spiralis*
14.	2015	Oborniki	wielkopolska	Nd	1	Nd	Nd	Nd	Nd	*T. britovi*
15.	2015	Piotrkowice	wielkopolska	32	2	6.25	10	None	Yes	*T. spiralis*
16.	2015	Szamotuły	wielkopolska	Nd	1	Nd	Nd	Nd	Nd	*T. spiralis*
17.	2016	Namysłów	opolskie	Nd	1	Nd	Nd	Nd	Nd	*T. spiralis*
18.	2016	Elbląg	warmińsko- mazurskie	Nd	1	Nd	Nd	Nd	Nd	*T. spiralis*
19.	2016	Choszczno	zachodnio- pomorskie	Nd	1	Nd	Nd	Nd	Nd	*T. spiralis*
20.	2016	Łobez	zachodnio- pomorskie	Nd	1	Nd	Nd	Nd	Nd	*T. spiralis*
21.	2017	Tczew	pomorskie	Nd	1	Nd	Nd	Nd	Nd	*T. spiralis*
22.	2017	Pelplin	pomorskie	366	11	3	50	None	Yes	*T. spiralis*
23.	2017	Bielsko-Biała	śląskie	Nd	1	Nd	Nd	Nd	Nd	*T. britovi*
24.	2017	Sławno	zachodnio- pomorskie	Nd	1	Nd	Nd	Nd	Nd	*T. spiralis*
25.	2018	Małkowo	pomorskie	101	47	46.5	None	None	No	*T. spiralis*
26.	2018	Dziećmiarki	wielkopolska	800	3	0.38	None	None	No	*T. spiralis*
27.	2019	Nowy Tomyśl	wielkopolska	2	2	100	3	3	Yes	*T. spiralis*
28.	2019	Chodzież	wielkopolska	343	18	5.2	7	None	Nd	*T. spiralis*
29.	2020	Wschowa	lubuskie	52	6	11.5	Nd	Nd	Nd	*T. spiralis*
30.	2020	Rosiny	zachodnio- pomorskie	115	10	8.9	Nd	Nd	No	*T. spiralis*

Nd—No data.

## Data Availability

Not applicable.
